# *BRCA* Variants Do Not Increase the Risk of Adverse Reactions in Patients With Ovarian Cancer: A Single-Center Real-World Study

**DOI:** 10.3389/fonc.2022.807748

**Published:** 2022-06-30

**Authors:** Kemin Li, Jing Zeng, Mengpei Zhang, Rutie Yin, Zhengyu Li

**Affiliations:** ^1^ The Department of Obstetrics and Gynecology, West China Second University Hospital of Sichuan University, Chengdu, China; ^2^ Key Laboratory of Birth Defects and Related Diseases of Women and Children (Sichuan University), Ministry of Education, Chengdu, China

**Keywords:** ovarian cancer, BRCA, myelosuppression, allergies to chemotherapy drugs, real-world study

## Abstract

**Objective:**

To study the correlation between *BRCA* mutation status and the risk of adverse reactions in patients with ovarian cancer.

**Method:**

A real-world study was conducted at the largest gynecological oncology center in western China, the West China Second University Hospital of Sichuan University. The research subjects were patients diagnosed with ovarian cancer who were initially treated in our hospital from January 2016 to January 2020 and had their *BRCA* gene status evaluated. Multivariate Cox analysis was conducted to investigate the correlation between the *BRCA* mutation status and adverse reactions in ovarian cancer patients during initial treatment.

**Results:**

A total of 349 ovarian cancer patients were enrolled, including 79 patients with pathogenic *BRCA* variants, resulting in a pathogenic mutation rate of 22.6%. Among these 79 patients, 57 had *BRCA1* variants and 22 had *BRCA2* variants, yielding a pathogenic mutation rate of 16.3% and 6.3%, respectively. Multivariate COX analysis revealed that pathogenic *BRCA* variants were not related to the risk of adverse reactions, such as myelosuppression and allergies to chemotherapy drugs (*P*>0.05), during the initial treatment of ovarian cancer.

**Conclusion:**

*BRCA* variants did not increase the risk of adverse reactions, such as myelosuppression and allergies to chemotherapy drugs, in ovarian cancer patients during initial treatment.

## Introduction

Ovarian cancer is the fifth leading cause of cancer deaths in women, ranking first among deaths related to gynecological cancer (1). In China, there are approximately 52,100 new cases of ovarian cancer and 22,500 deaths every year (2). Platinum-based chemotherapy remains the first-line treatment for ovarian cancer following satisfactory surgical cytoreduction. Chemotherapy drugs cause various types and degrees of adverse reactions (3). For example, myelosuppression is one of the most common adverse reactions to chemotherapy for ovarian cancer. Studies have shown that among the newly diagnosed advanced ovarian cancer patients treated with paclitaxel (PTX) and carboplatin plus bevacizumab, roughly 12% suffer from Grade 3-4 anemia, and as high as 21% of the patients had neutropenia (4).

Homologous recombination repair (HRR) involves DNA double-strand break (DSB) repair and maintenance of genetic stability, deficiencies in which may lead to malignancies. Studies have shown that up to 50% of high-grade serous ovarian cancers (HGSOC) are related to HRR deficiency and express *BRCA1* or *BRCA2* variants (5). Germline and somatic HRR mutations are highly predictive of primary platinum sensitivity and improvement in overall survival (OS). As reported, the median OS of carriers of germline mutations involved in HRR is 66 months, that of carriers of somatic mutations is 59 months, and that of those without HRR-related mutations is 41 months (6). It has rarely been described whether the *BRCA* mutation status affects the risk of adverse reactions caused by chemotherapy in ovarian cancer patients, and the conclusions are inconsistent. Kotsopoulos et al. (7) reported that *BRCA* variants do not increase the risk of hematological adverse reactions caused by chemotherapy in patients with ovarian cancer; Rolfe et al. (8) reported that *BRCA* variants do not increase the risk of allergic reactions to carboplatin; whereas, Giannone et al. (9) found that the allergic reactions to carboplatin in patients with *BRCA* mutations are significantly increased during chemotherapy.

In the present study, using real-world data, we performed a multi-factor analysis of the correlation between the BRCA mutation status of patients with ovarian cancer and the risk of adverse reactions during initial treatment, providing a basis for the standardized management of adverse reactions in these patients.

## Materials and Methods

The West China Second University Hospital of Sichuan University (WCSUH) is the largest gynecological oncology diagnostic and treatment center in western China. The patients diagnosed with ovarian cancer who had their initial treatment at our hospital from January 2016 to January 2020 were the research subjects, all of whom were tested for *BRCA* mutation status. There were no restrictions on the age of the research subjects or the pathological type of ovarian cancer. No patients had received radiotherapy, chemotherapy, or other anti-tumor therapy, nor did they have any cancer history of other systems. Real-world clinical parameters of the research subjects who met the enrollment criteria were collected, including age, pathological type, neoadjuvant chemotherapy, *BRCA* mutation, FIGO (the International Federation of Gynecology and Obstetrics) staging, adverse reactions during initial treatment, family history of cancer, and granulocyte colony-stimulating factor (G-CSF) application during treatment.

Genetic testing was performed by the well-known international genetics company BGI Group. The germline mutation status of *BRCA1* and *BRCA1* was tested using patient’s blood samples. Target capture combined with second-generation high-through put sequencing technology was used to analyze the variations (including point mutations, deletions, and insertions within 20 bp) in the related gene exons and the adjacent ± 10 bp introns, yielding pathogenic *BRCA* variants, *BRCA* variants of unknown significance (VUS), likely benign *BRCA* variants and benign *BRCA* variants. The pathogenic *BRCA* variants are noted as *BRCA* (+), while *BRCA* VUS and likely benign and benign *BRCA* variants are grouped into *BRCA* (–).

All patients received standard treatment following the FIGO treatment guidelines for ovarian cancer at the time of treatment. After the initial treatment, maintenance therapy, clinical observation, or clinical trials were provided according to the patient’s preference, and all patients had lifetime follow-up.

Patient adverse reactions were defined according to the Common Terminology Criteria for Adverse Events (CTCAE), including low white blood cell count (leukopenia), low neutrophil count (neutropenia), low platelet count (thrombocytopenia), anemia, thromboembolism, and allergies to chemotherapy drugs (platinum or PTX).

Data are expressed as the mean ± standard deviation. The multivariate COX model was employed to analyze the correlation between pathogenic *BRCA* variants and various adverse reactions in patients with ovarian cancer during initial treatment. The factors include age, stage, regimens, cycles, neoadjuvant chemotherapy, surgical outcome (R0/R1/R2), thrombosis, family history of cancer, pathological type, adverse reactions (leukopenia, neutropenia, thrombocytopenia, anemia, thromboembolism, and allergies to chemotherapy drugs) and BRCA. *P*<0.05 was considered statistically significant.

## Results

### Basic Characteristics of Research Subjects

The basic characteristics of the study subjects are shown in **Table 1**. A total of 349 patients with ovarian cancer who met the criteria were included. All patients received paclitaxel + cisplatin/carboplatin. The mean age was 50.0 ± 13.1 years old and 333 cases (95.4%) were ovarian epithelial cancer. According to FIGO staging, there were 68 cases (19.5%) of Stage IA-IIA and 281 cases (80.5%) of Stage ≥IIB. A total of 79 patients carried pathogenic *BRCA* variants, which is a pathogenic mutation rate of 22.6%. Among these, 57 patients carried *BRCA1* variants and 22 carried *BRCA2* variants, yielding a pathogenic mutation rate of 16.3% and 6.3%, respectively. There were 175 patients with a family history of cancer.

**Table 1:** Basic characteristics of research subjects.

		BRCA+					
		BRCA1+	BRCA2+	total	BRCA-	total	P
n		57	22	79	270	349	
Years		49.6±13.5	50.6±13.2	50.1±13.3	49.8±12.9	50.0±13.1	0.75
Neoadjuvant chemotherapy		17	10	27	58	85	0.02
Family history		43	14	57	118	175	<0.0001
Pathological type	Epithelial	52	21	73	260	333	0.15
	carcinosarcoma	3	1	4	3	7	0.04
	others	2	0	2	7	9	0.98
FIGO stage	IA-IIA	5	0	5	63	68	0.002
	≥IIB	52	22	74	207	281	0.002
Chemotherapy cycles (mean)	NC	0.8	1.7	1.0	1.0	1.0	0.89
	AC	7.4	7.0	7.4	7.4	7.4	0.79
	total	8.2	8.7	8.8	8.4	8.4	0.86
Regimens	T+P	12	4	16	49	65	0.67
	T+C	10	4	14	45	59	0.83
	T+P/C	35	14	49	176	225	0.61
White blood cell	≥1 grade	57	22	79	263	342	0.3
	≥3 grade	15	6	21	92	113	0.21
neutrophils	≥1 grade	57	22	79	265	344	0.42
	≥3 grade	14	9	23	118	141	0.02
Anemia	≥1 grade	53	21	74	247	321	0.53
	≥3 grade	0	0	0	2	2	0.8
platelets	≥1 grade	31	6	37	106	143	0.23
	≥3 grade	6	1	7	28	35	0.69
thromboembolism		11	2	12	39	52	0.87
allergy	platinum	10	1	11	15	26	0.02
	Taxol	2	1	3	8	11	0.71
G-CSF		37	11	48	161	209	0.86

G-CSF, granulocyte colony stimulating factor; BRCA, Breast Cancer susceptibility gene; NC, neoadjuvant chemotherapy; AC, adjuvant chemotherapy; T, paclitaxel; P, cisplatin; C, carboplatin.

### Occurrences of *BRCA* Variants and Adverse Reactions

The occurrences of *BRCA* variants and adverse reactions are shown in **Table 1**. Among the *BRCA* (+) patients, 21 (26.6%), 0 (0%), and 7 (8.9%) patients with CTCAE ≥ Grade 3 experienced leukopenia, anemia, and thrombocytopenia, respectively, which is clearly less than *BRCA* (–) patients whose corresponding values were 113 (41.9%), 2 (0.2%), and 35 (13.0%). Nevertheless, the differences were not statistically significant(*P*>0.05).

Among the *BRCA* (+) patients, 23 (29.1%) suffered from CTCAE ≥ Grade 3 neutropenia, which is less than 118 (43.7%) of the BRCA (–) patients. The difference was statistically significant (P <0.05). A total of 12 (15.2%) *BRCA* (+) patients suffered from thromboembolism, which is less than 52 (19.3%) of the *BRCA* (–) patients; however, the difference was not statistically significant (*P*>0.05). Moreover, a total of 48 (60.8%) *BRCA* (+) patients were administered G-CSF due to myelosuppression, which is much less than 209 (77.4%) of the *BRCA* (–) patients; however, the difference was not statistically significant (*P*>0.05).

Platinum-based chemotherapeutic allergy occurred in 11 (13.9%) *BRCA* (+) patients, which is significantly more than 15 (5.6%) of the *BRCA* (–) patients. This difference was statistically significant (*P*<0.05). Among the *BRCA* (+) patients, 3 (3.8%) developed an allergy to PTX, while 8 (3.0%) of the *BRCA* (–) patients developed a PTX allergy; however, the difference was not statistically significant (*P*>0.05).

### Correlation Between Pathogenic *BRCA* Variants and Adverse Reactions

The correlation between pathogenic *BRCA* variants and adverse reactions is shown in **Table 2**. According to the multivariate COX model, after considering other factors (age, stage, regimens, cycles, neoadjuvant chemotherapy, surgical outcome (R0/R1/R2), thrombosis, family history of cancer, pathological type), pathogenic *BRCA* variants (including *BRCA1* and *BRCA2*) were not correlated with the occurrence of adverse reactions during the initial treatment of ovarian cancer (*P*>0.05). The adverse reactions included leukopenia, neutropenia, thrombocytopenia, anemia, thromboembolism, and allergies to chemotherapy drugs (platinum or PTX).

**Table 2:** Correlation between pathogenic *BRCA* variants and adverse reactions.

Adverse reactions	genes	Standard error	Wald	P VALUE	95%CI
Allergic reaction	BRCA1	177.6	0.006	0.9382	(0.887-1.228)
	BRCA2	110.9	0.0664	0.7967	(0.793-1.156
Abnormal liver function	BRCA1	0.4873	0.3328	0.564	(0.540-1.226)
	BRCA2	0.7201	0.5238	0.4692	(0.472-2.367)
leukopenia	BRCA1	0.4717	0.3209	0.5711	(0.558-2.154)
	BRCA2	0.8832	2.462	0.1166	(0.332-3.441)
neutropenia	BRCA1	0.4602	0.1066	0.744	(0.792-1.164)
	BRCA2	1.0135	1.8695	0.1715	(0.223-3.471)
anemia	BRCA1	0.4993	0.1056	0.7452	(0.669-1.132)
	BRCA2	0.8745	0.089	0.7654	(0.693-1.201)
thrombocytopenia	BRCA1	0.5124	1.695	0.1929	(0.341-2.998)
	BRCA2	0.9958	0.0635	0.8011	(0.889-1.219)
thromboembolism	BRCA1	0.7396	0.0029	0.9571	(0.993-1.077)
	BRCA2	77.5065	0.0012	0.9725	(0.989-1.041)

BRCA, Breast Cancer susceptibility gene.

## Discussion

PARP is a key enzyme responsible for repairing DNA single-strand breaks (SSB), inhibition of which leads to persistent SSB that transform into severe double-strand breaks (DSB) during DNA replication. During the process of cell division, DSB in normal cells can be effectively repaired by homologous recombination repair (HRR). Tumors displaying HRR deficiency, such as ovarian cancer in patients with *BRCA1*/2 variants, cannot accurately repair DNA damage, and accumulation of such damage may lead to cell death. In the same manner, tumor patients carrying germline *BRCA1*/2 variants cannot accurately repair DNA damage following the administration of chemotherapy drugs and similar anti-tumor treatments, and as a result, may also have increased risk or elevated severity of adverse reactions. Should the occurrence of adverse reactions, especially ≥ Grade 3 adverse reactions, significantly increase during chemotherapy in patients with pathogenic *BRCA* variants, then treating pathogenic *BRCA* variant carriers as high-risk patients and improving the standard treatment management would reduce the impact of adverse reactions on patients and improve their quality of life.

Hematological toxicity, such as myelosuppression, is one of the most common adverse reactions to chemotherapy. Whether *BRCA* variants increase the occurrence of myelosuppression in patients undergoing chemotherapy remains controversial. In their study on the toxicity of PTX-based chemotherapy in pathogenic *BRCA* variant carriers with breast cancer, Bayraktar et al. (10) analyzed the hematological toxicity during PTX chemotherapy in a total of 790 patients of various *BRCA* mutation statuses and found that the occurrence of anemia and leukopenia in *BRCA* (–) patients was significantly higher than that in pathogenic *BRCA* variant carriers. Moreover, pathogenic *BRCA2* variants were a predictor of hematological adverse reactions in breast cancer patients during PTX chemotherapy. However, Kotsopoulos *et al.* (7) revealed that pathogenic *BRCA* variants are not related to the hematological toxicity caused by platinum-based combination chemotherapy in ovarian cancer patients. A total of 432 ovarian cancer patients were enrolled, including 130 *BRCA* (+) and 302 *BRCA* (–). The results demonstrated that the risk of neutropenia, anemia, and thrombocytopenia in patients with pathogenic *BRCA* variants did not differ from that in the *BRCA* (–) patients. Using real-world data, the present study shows that the occurrence of myelosuppression (including leukopenia, neutropenia, thrombocytopenia, and anemia) during initial treatment in ovarian cancer patients with pathogenic *BRCA* variants was substantially lower than that in BRCA (–) patients; nevertheless, the multivariate analysis showed no significant correlation between pathogenic *BRCA* variants and the occurrence of myelosuppression.

Infusion reactions and even severe allergic reactions may occur during or after chemotherapy. Whether *BRCA* variants increase the occurrence of allergic reactions to chemotherapy drugs remains in conclusive. Rolfe et al. (8) studied the relationship between *BRCA* mutation status and the risk of carboplatin-related allergic reactions and found that pathogenic *BRCA* variants did not increase the risk of carboplatin allergy in patients with ovarian cancer. However, Giannone (9) et al. systematically evaluated the relationship between pathogenic *BRCA* variants and the risk of carboplatin-related allergic reactions, reviewing a total of 5 studies including 432 patients with ovarian cancer, and found that pathogenic *BRCA* variants significantly increased carboplatin allergy risk in patients with ovarian cancer. The results of our study demonstrate that the occurrence of carboplatin allergic reactions during chemotherapy in ovarian cancer patients with pathogenic *BRCA* variants was substantially increased; however, the multivariate COX analysis did not show statistical significance.

The present study also analyzed whether pathogenic *BRCA* variants increase the risk of thromboembolism and G-CSF application in patients with ovarian cancer during the initial treatment, and reached a negative conclusion. At present, there are few relevant studies and the ratio of patients with allergic reactions in the present study was low; therefore, the reliability of the conclusions needs to be tested further.

Our study was a single-center study with a small sample size, and the low occurrence of some adverse reactions, such as carboplatin and PTX allergies, may affect the reliability of the conclusions. However, so far, the present study is the largest study of real-world data, and considered many confounding factors, such as neoadjuvant chemotherapy, family history of cancer, and pathological tumor type, and adopted multi-factor analysis to avoid the influence of other factors. Moreover, the adopted real-world data truly represents actual clinical practice and has great value for clinical guidance.

## Conclusion

*BRCA* variants did not increase the risk of adverse reactions, such as myelosuppression and allergies to chemotherapy drugs, in ovarian cancer patients during initial treatment.

## Data Availability Statement

The original contributions presented in the study are included in the article/supplementary materials. Further inquiries can be directed to the corresponding authors.

## Ethics Statement

The studies involving human participants were reviewed and approved by Approvalled Medical Ethics Committee of West China Second University Hospital, Sichuan University. Ethical Lot Number:20200076. Written informed consent for participation was not required for this study in accordance with the national legislation and the institutional requirements.

## Author Contributions

Conception: KL, RY. Design: all authors. Data collection: KL, MZ. Data analysis: KL, JZ. Manuscript writing: KL, MZ. All authors contributed to the article and approved the submitted version.

## Conflict of Interest

The authors declare that the research was conducted in the absence of any commercial or financial relationships that could be construed as a potential conflict of interest.

## Publisher’s Note

All claims expressed in this article are solely those of the authors and do not necessarily represent those of their affiliated organizations, or those of the publisher, the editors and the reviewers. Any product that may be evaluated in this article, or claim that may be made by its manufacturer, is not guaranteed or endorsed by the publisher.

## Abbreviations

BRCA, breast cancer susceptibility gene; PTX, paclitaxel; HRR, Homologous recombination repair; DSB, DNA double-strand break; OS, overall survival; HGSOC, high-grade serous ovarian cancers; FIGO, the International Federation of Gynecology and Obstetrics; G-CSF, granulocyte colony-stimulating factor; VUS, variants of unknown significance; CTCAE, Common Terminology Criteria for Adverse Events; AE, Adverse Events; PARP, poly ADP-ribose polymerase.
